# Overdominance for fitness: a genomic comparison between empirical and simulated data with *Drosophila melanogaster*

**DOI:** 10.1093/genetics/iyag050

**Published:** 2026-02-24

**Authors:** Inés González-Castellano, Humberto Quesada, Sebastián Ramos-Onsins, Aurora García-Dorado, Armando Caballero

**Affiliations:** Centro de Investigación Mariña, Universidade de Vigo, Vigo 36310, Spain; Universidade da Coruña, A Coruña 15008, Spain; Centro de Investigación Mariña, Universidade de Vigo, Vigo 36310, Spain; Centre for Research in Agricultural Genomics CRAG (CSIC-IRTA-UAB-UB), Cerdanyola del Vallès, Barcelona 08193, Spain; Departamento de Genética, Facultad de Ciencias Biológicas, Universidad Complutense, Madrid 28040, Spain; Centro de Investigación Mariña, Universidade de Vigo, Vigo 36310, Spain

**Keywords:** heterozygote advantage, balancing selection, nucleotide diversity, inbreeding load, computer simulations, SNP

## Abstract

Overdominance (heterozygote advantage) for fitness is a form of balancing selection which supports the maintenance of genetic polymorphisms in the populations. This mode of selection is expected to generate a conspicuous footprint on neutral genetic variation in genomic regions of restricted recombination, which contrasts with the signature generated by other types of natural selection. In particular, gene diversity is expected to be increased at neutral sites tightly linked to overdominant sites, as opposed to background selection and selective sweeps. We produced extensive whole-genome sequencing data to analyze genetic diversity across regions exhibiting varying recombination patterns in a *Drosophila melanogaster* population with known demographic history. The results were compared with simulation data in order to quantify the magnitude of the contribution of overdominant loci to genetic diversity which could be compatible with observations. We analyzed sequencing data from 51 individual male flies sampled from a large population and estimated the average nucleotide diversity (*π*) in 100-kb consecutive windows across the main autosomal chromosomes. By using the available recombination map in the species, we evaluated the changes in *π* in relation with the levels of recombination across genomic regions. We then carried out computer simulations following the demographic history, the genomic architecture and the recombination map of the main autosomal chromosomes assuming models which include deleterious (background selection) and advantageous (selective sweeps) mutations, or models also including increasing rates of overdominant mutations. By comparing the results obtained from simulations and the observed data, we conclude that a parsimonious model of background selection and adaptation to captivity explains the observed patterns of neutral variation in the studied population better than the models including overdominance.

## Introduction

It is well known that selection shapes genomic variation at neutral sites linked to selective ones, the so-called linked selection hypothesis (Cutter and Payseur 2013; Elyashiv et al. 2016). Thus, purifying selection against deleterious variants (background selection) and the spreading of beneficial mutations toward fixation (selective sweeps) reduce nearby neutral diversity (Maynard-Smith and Haigh 1974; Berry et al. 1991; Charlesworth et al. 1993). Because lower genetic recombination implies tighter linkage, the effects of these types of selection on neutral linked variants are amplified in regions of low recombination, leading to the positive correlation between recombination rates and genetic diversity that has been reported in numerous species (Begun and Aquadro 1992; Charlesworth and Campos 2014; Comeron 2014, 2017). However, heterozygote advantage for fitness, also known as overdominance, is a form of balancing selection that maintains alleles in populations as polymorphisms with stable frequencies (Kimura and Ohta 1971). Furthermore, in gametes bearing different alleles at an overdominant site, the divergence at neutral sites is inversely related to 2*N_e_c*, where *N_e_* is the effective population size (Wright 1931) and *c* is the recombination fraction between the neutral and the overdominant sites (Hudson 1990). Therefore, gene diversity is expected to be increased at neutral sites tightly linked to overdominant sites, as opposed to background selection and selective sweeps. Thus, the resulting signature of overdominance is that neutral variants linked to the balanced sites are also kept at intermediate frequencies in the populations, what is known as associative overdominance (Frydenberg 1963; Pamilo and Pálsson 1998; Comeron 2014; Bersabé et al. 2016; Zhao and Charlesworth 2016), generating an excess of neutral diversity relative to that expected in the absence of selection (Nielsen 2005; Charlesworth 2006).

True overdominance appears to be a rather rare genetic condition with only a few well-documented cases, such as sickle cell anemia (Allison 1954), immunity-related genes in humans (Bitarello et al. 2018; Minias and Vinkler 2022), warfarin resistance in rats (Partridge 1979), female fecundity in sheep (Gemmell and Slate 2006), male horn size in sheep (Johnston et al. 2013), or productivity in *Caenorhabditis elegans* (Peters et al. 2003), among other examples in different species (see review by Hedrick 2012). Overdominance can be generated by antagonistic pleiotropy affecting different fitness traits (Rose 1982; Roff 1997; Fernández et al. 2005), for instance, due to a negative relationship between reproduction and lifespan, as observed in many vertebrates (Lemaître et al. 2015). In humans, an excess of genetic variants with opposing effects on early-onset and late-onset human diseases has also been found using genetic data (Rodríguez et al. 2017). In addition, genetic variants that have been favored in the past may not be beneficial for some traits in the current environment, also generating antagonistic pleiotropy (Corbett et al. 2018). An extensive meta-analysis of selection coefficients reported in the literature from natural populations of multiple taxa found that about 2% of the coefficients examined showed overdominant behavior (Thurman and Barrett 2016). This figure should be interpreted with caution due to the low statistical precision of many estimates (Thurman and Barrett 2016) and given that most genes showing heterozygous advantage in vertebrates were those classically known to be the paradigm of balancing selection, such as the *β-hemoglobin* and the major histocompatibility complex (*MHC*)/human leukocyte antigen (*HLA*) genes. Thus, the role of overdominance in maintaining variation in the genome is expected to be restricted to particular genes and mostly confined to low-recombining regions (Draghi and Whitlock 2015; Fijarczyk and Babik 2015).

It should be noted that associative overdominance in regions of low recombination can arise not only from true overdominance but also from complementation between recessive deleterious mutations at closely linked loci in repulsion phase, what is known as pseudo-overdominance (Ohta 1971; Becher et al. 2020; Gilbert et al. 2020; Waller 2021; Abu-Awad and Waller 2023). The deleterious effects of haplotypes are masked in heterozygous condition, so that the heterozygotes appear to have the highest fitness, which can be mistaken for true overdominance (Waller 2021). Because pseudo-overdominance mimics balancing selection, it allows neutral diversity to be maintained in regions of low recombination (Zhao and Charlesworth 2016; Gilbert et al. 2020; Waller 2021; Sianta et al. 2023), which may be crucial for small populations (Schou et al. 2017).

An excess of genetic variance has been found for different fitness traits relative to expectations from mutation-selection balance (Charlesworth 2015; Sharp and Agrawal 2018), which could be ascribed to balancing selection. Different studies have also shown that genomic regions subjected to balancing selection can be common (Andrés et al. 2009; Gao et al. 2015; Schou et al. 2017; Siewert and Voight 2017; Bitarello et al. 2018; Wang et al. 2019; Gilbert et al. 2020; Soni et al. 2022). These episodes of balancing selection could be caused by pure overdominance, pseudo-overdominance, or other forms of balancing selection, such as frequency-dependent selection, selection pressures varying in space or time, or genotype–environment interactions (Lande 1976; Rose 1982; García-Dorado 1986; Mukai 1988; Roff 1997; Santos 1997; Charlesworth and Hughes 1999; Charlesworth 2006).

The detection of signatures of balancing selection, through a pattern of excess of common polymorphisms, can be made by different statistics, classically by Tajima’s (1989)  *D* or the HKA test (Hudson et al. 1987). Nonetheless, these methods have a low power and tend to be extremely sensitive to nonequilibrium demography or population structure (Fijarczyk and Babik 2015; Siewert and Voight 2017). More powerful statistics have recently revealed some evidence of balancing selection in the human genome. For example, Andrés et al. (2009) detected 60 genes with significant signatures of long-term balancing selection as shown by their excess of polymorphism and intermediate-frequency alleles. Likewise, Bitarello et al. (2018) indicated that long-term balancing selection in humans may be shaping variation in up to 2% of variable genomic positions. Moreover, Soni et al. (2022) provided evidence that hundreds of nonsynonymous polymorphisms in the human genome could be subject to balancing selection. However, all these evidences of balancing selection may be due to sources different from overdominance for fitness, as mentioned earlier, except perhaps for particular genomic regions, so it is unclear whether or not overdominance is a widespread phenomenon.

In addition to its effects on genetic diversity, overdominance has also been discussed in terms of its role on inbreeding depression, i.e. the decline in fitness-related traits observed in inbred individuals compared to their non-inbred counterparts (Keller and Waller 2002; Hasselgren and Norén 2019). Inbreeding increases genomic homozygosity, allowing the expression of the inbreeding load (*B*), which is the detrimental genetic burden that is concealed in the heterozygous state in outbred populations (Morton et al. 1956). This load can be attributed to the exposure of (partially) recessive deleterious alleles in homozygotes (dominance hypothesis) or to the homozygosity of loci with heterozygote advantage (overdominance hypothesis). A recent simulation study shows that the usual methods to estimate inbreeding depression from dominant loci are also applicable and precisely estimate inbreeding depression from overdominant loci (González-Castellano et al. 2025b). The relative contribution of these 2 sources of inbreeding load has been debated for decades (Ayroles et al. 2009; Charlesworth and Willis 2009; Paige 2010; Schou et al. 2018), although the current consensus is that partial dominance plays the major role in inbreeding depression, as supported by the majority of empirical and theoretical studies (Charlesworth and Charlesworth 1987; 1999; Barrett and Charlesworth 1991; Crow 1999; Roff 2002; Hedrick 2012; Yang et al. 2017; González-Castellano et al. 2025a).

In this study, we set out to elucidate the possible contribution of overdominance to genetic diversity by contrasting genomic data with simulation results. We analyzed whole-genome sequencing data from a laboratory population of *Drosophila melanogaster* with known demographic history to evaluate patterns of variability across genomic regions with contrasting recombination rates. The unusual circumstance that the recent demography of the population is known allowed us to develop models to investigate the genetic architecture of the population. Thus, we performed computer simulations based on the population demography, genetic architecture, and recombination map of the species, considering only neutral, deleterious, and additive advantageous mutations or also adding an increasing number of overdominant mutations, in order to assess whether the empirical data are compatible with the absence or presence of overdominance. We do not consider in the simulations other possible types of balancing selection, although pseudo-overdominance is also implicit in the models.

## Materials and methods

### Experimental population

A *D. melanogaster* population was founded in 2009 from a wine cellar population and maintained with around 2,800 individuals as described by López-Cortegano et al. (2016) and Pérez-Pereira et al. (2021). The population was maintained with constant temperature (25 °C) and continuous lighting with circular mixing of 32 bottles (numbered from 1 to 32) each with 40–50 individuals of each sex and the feeding medium used in our laboratory (1 L water, 200 g brewer's yeast, 50 g sucrose, 12 g agar, 2.5 g NaCl, and 5 ml propionic acid). In each generation, 20–25 individuals (about half of each sex) were taken from bottle *i* and the same number from bottle *i*–1 (from bottle 32 in the case *i* = 1) to found bottle *i* of the following generation. Male and female adults in each bottle were removed after a week, before the next generation emerged, to avoid overlapping generations. At generation 208, after ∼10 yr of maintenance in the laboratory with an approximately constant population size, a total of 51 males were sampled from the population to carry out individual whole-genome sequencing.

### DNA extraction, sequencing, and SNP calling

The sampled individuals were frozen in liquid nitrogen and stored at −80 °C. An optimized protocol for DNA extraction from individual flies including treatment with RNase was carried out with the Gentra Puregene Cell Kit (Qiagen). Whole-genome sequencing of 2 × 150 bp paired-end was performed by Macrogen (South Korea) on an Illumina Novaseq 6000 instrument using Nextera XT DNA libraries. FASTQ sequencing files underwent a first quality control with FastQC (Andrews 2010), and adapters were removed with Trimmomatic (Bolger et al. 2014) using Nextera adapter list. ERNE-FILTER v2 (Del Fabbro et al. 2013) was used to carry out quality and size trimming (minimum sequence length after trimming = 36). After a new quality control with FastQC, the obtained reads were mapped against the 6.14 *D. melanogaster* reference genome with BWA-MEM (Li, 2013). The resulting SAM files were converted to indexed BAM files with SAMtools v1 (Danecek et al. 2021), and chromosomes were isolated and indexed. SAMtools v1 was also used for PCR duplicate removal and filtering (minimum mapping quality = 20). Qualimap v2 (Okonechnikov et al. 2016) was used to perform the quality control of the alignment. The average sequencing depth and mapping quality were 43.49× (±1.19) and 58.38 (±0.01), respectively. Raw variant calling (minimum phred-scaled confidence threshold = 10) was carried out using the HaplotypeCaller tool from GATK v4 (Van der Auwera and O’Connor 2020). The resulting gVCF files were combined using the GATK CombineGVCFs tool, keeping only biallelic SNPs. Highly repeated sites, low information regions, and SNPs within 10 bp of an indel were removed with BCFtools (Danecek et al. 2021). Finally, SNPs were also filtered with the GATK VariantFiltration tool applying the recommended presets. The final number of called SNPs was 1,541,774. Various filters were then applied to remove potentially selective SNPs. First, ANNOVAR v4.19 (Wang et al. 2010) was used to annotate SNPs into categories such as 5′UTR and 3′UTR, synonymous, missense (nonsynonymous), and loss-of-function (LoF), which includes stop-gain and stop-loss mutations. Non-synonymous, LoF and UTR SNPs were excluded from the dataset. Following this, intergenic regions in the UCSC genome browser conservation 124-way track (insects) (https://genome.ucsc.edu/cgi-bin/hgTrackUi?db=dm6&g=cons124way) with phastCons scores ≥0.80 (Zheng and Zhao 2022) were also removed (https://genome.ucsc.edu/cgi-bin/hgTrackUi?hgsid=2362308529_vdHqtNjHfKu9bHbkfr0aKlX5a2he&g=cons124way&hgTracksConfigPage=configure). After applying all filters, 1,365,568 SNPs remained in the final dataset.

### Genetic map usage

We used the average recombination rate (*c*) of 100-kb genomic windows reported in the *D. melanogaster* genetic map for autosomes obtained by Comeron et al. (2012). Since this map represents genetic distances in centimorgans (cM) per megabase (Mb) per female meiosis and given that Drosophila males do not undergo recombination, we halved these recombination rates. In addition, since Comeron et al.’s (2012) genetic map refers to the dm5 version, we first converted its coordinates from dm5 to dm6 using the Flybase Coordinates Converter tool (Gramates et al. 2022). Called SNPs were grouped in 100-kb windows, and those windows for which the recombination rate was not reported in Comeron et al.’s (2012) map were discarded, such that a total number of 1,356,231 SNPs in 965 windows of 100 kb were available for analysis. In particular, 395,115 SNPs in chromosome arm 2L (230 windows), 288,678 SNPs in chromosome arm 2R (211 windows), 341,012 SNPs in chromosome arm 3L (245 windows), and 331,426 SNPs in chromosome arm 3R (279 windows). Nucleotide diversity (Nei and Tajima 1981) in the sampled individuals was obtained with VCFtools (Danecek et al. 2011) for each 100-kb window (${\bar{\pi }}_{\mathrm{window}}$). Linkage disequilibrium statistic *r*^2^ between consecutive SNPs was obtained using PLINK (Purcell et al. 2007).

### Estimation of effective population size (*N_e_*) from linkage disequilibrium between SNPs using the software GONE

A previous estimate of the effective size (*N_e_*) of the laboratory population in the most recent generations was obtained using a subset of 17 males, being of the order of 1,000 individuals (Novo et al. 2023b). In the present study, a more comprehensive estimation of historical *N_e_* (going back 300 generations in the past) was obtained with the software GONE (Santiago et al. 2020; available at https://github.com/esrud/GONE) using the whole set of 51 individuals. We used a set of SNPs filtering out those with a high linkage disequilibrium (*r*^2^ > 0.2) using the command *–indep-pairwise 100 20 0.20* of PLINK1.9 (Purcell et al. 2007), to obtain a total of 62,147 SNPs. For an accurate estimation of historical *N_e_*, a precise genetic map is required. As explained in the previous section, the genetic distances were obtained from the *D. melanogaster* genetic map (Comeron et al. 2012), corrected for the lack of recombination in males (Novo et al. 2023b). The estimation by GONE assumed the default options of the software: unknown phase, Haldane’s correction for genetic distances, no minor allele frequency pruning, use of all SNPs including those with missing data, and windows with a maximum value of recombination frequency of 0.05.

### Estimation of effective population size (*N_e_*) from the spectrum of allele frequencies with the software StairwayPlot2

An estimation of historical *N_e_* was also obtained with the software StairwayPlot2 (Liu and Fu, 2020; available at https://github.com/xiaoming-liu/stairway-plot-v2), from the allele frequency spectrum of both variant and invariant sites sequenced in the 51 samples. Starting from the initial VCF file containing all detected SNPs and considering all sequenced invariant positions, we computed the counts and folded frequencies of these sites, resulting in the folded site frequency spectrum (fSFS). The fSFS was calculated separately for total, silent, synonymous, and nonsynonymous sites.

To obtain these spectra, we used a custom pipeline based on the mstatspop software (https://github.com/CRAGENOMICA/mstatspop) to generate the fSFS for each chromosome. The pipeline first converts the VCF file to transposed FASTA (TFA) format using gVCF2TFasta (https://github.com/sramosonsins/gVCF2tFasta). Prior to running mstatspop, silent and synonymous sites were identified following the method of Nei and Gojobori (1986) using the software fastaconvtr (https://github.com/CRAGENOMICA/fastaconvtr).

The genome-wide fSFS was obtained by summing the chromosome-specific fSFS using an R script. Chromosome 2L was excluded from genome-wide analyses because it contains large inverted regions that distort the fSFS. The fSFS was obtained for each chromosome and for the whole genome (hereafter always excluding chromosome 2L). Estimates of genomic variability were also obtained for total, silent, synonymous, and nonsynonymous sites.

Genome-wide fSFS derived from silent (74,577,602) and synonymous (3,316,854) sites were used as input for StairwayPlot2 to infer demographic history, as these site categories are expected to evolve largely neutrally. We assumed a mutation rate of *u* = 3 × 10^−9^ mutations per site per generation (Wang et al. 2023) and 20 generations per year to estimate *N_e_* through time.

### Computer simulations

We used the SLiM4 software (Haller and Messer 2023) to carry out forward-in-time simulations of a panmictic population of *N* = 10,000 diploid individuals for up to 50,000 discrete generations under the action of mutation, recombination, selection, and drift. This ancestral effective size is justified by the estimated historical *N_e_* obtained from the empirical data by the software GONE (shown below). Where noted, we carried out additional simulations considering an ancestral population size 10 times larger (*N* = 100,000 individuals). In order to emulate the capture from nature and maintenance in the laboratory of the experimental *Drosophila* population at its estimated effective population size, at generation 49,800 the ancestral population was reduced in size to 1,000 individuals (as deduced from GONE estimates of recent *N_e_*) and divided into 32 subpopulations of 32 individuals each that were simulated for 200 additional generations. During these last 200 generations of the simulations, a circular migration of 50% of the individuals between the subpopulations was established, mimicking the circular mixing between the 32 bottles carried out in the laboratory. In the last generation, 1 or 2 individuals from each small subpopulation were sampled (like in the experimental design), so that the genomes of 51 individuals were analyzed, corresponding to the 51 flies sequenced to obtain the empirical data.

We carried out simulations for each of the 4 autosomal arms of *D. melanogaster* separately (2L, 2R, 3L, and 3R), using Comeron et al.’s (2012) genetic map and the same corresponding adjacent 100-kb windows as for the empirical data. To closely mimic the genomic architecture of the chromosome arms, we differentiated genic (about 40% of the autosomal genome) and nongenic regions along the simulated sequences. The positions of genes were extracted from the dm5 annotated sequence of each arm available in GenBank, accession numbers AE014134.5 (chromosome arm 2L), AE013599.4 (2R), AE014296.4 (3L), and AE014297.2 (3R). Gene conversion (GC) was assumed to occur at a rate of 2 × 10^−8^ per nucleotide per generation, randomly distributed across the genome with tract lengths of 441 bp, following the results of Miller et al. (2016).

The parameters considered for the standard model M1 are described first. In each simulation, mutations were assumed to occur at a rate higher than typical empirical estimates, using 1.6 × 10^−8^ per nucleotide per generation to account for a realistic overall mutation rate. This rate enabled us to simulate the intended number of deleterious mutations and a sufficiently large number of neutral SNPs for analysis. Table 1 shows the mutation rates and effects and dominance of mutations for the different models investigated. Deleterious mutations (slightly or strongly deleterious) were assumed to occur in genic regions, in a two-thirds proportion, whereas the remainder one-third were assumed to be neutral. For each mutation with fitness effect, fitness values of 1, 1 + *sh*, and 1 + *s* were assumed for the wild-type homozygote, the heterozygote, and the mutant homozygote, respectively. The fitness of each individual was assumed to be multiplicative across loci, as usually assumed for fitness (Caballero 2020, p. 161).

**Table 1.** Mutational parameters assumed in the simulations without overdominant mutations.

Model	*U_neu_*	*U_del_*	*s_del_*	*h_del_*	*U_sdel_*	*s_sdel_*	*h_sdel_*	*U_let_*	*h_let_*	*U_adap_*	*s_adap_*	*h_adap_*
M1	0.274	0.049	−0.2	0.283	0.06	−0.0001	0.5	0.003	0.02	0	—	—
M1S^a^	0.273	0.049	−0.2	0.283	0.06	−0.0001	0.5	0.003	0.02	0.0009	0.05	0.5
M2	0.274	0.049	−0.2	0.283	0.06	−0.0005	0.5	0.003	0.02	0	—	—
M2S	0.273	0.049	−0.2	0.283	0.06	−0.0005	0.5	0.003	0.02	0.0009	0.05	0.5
M3	0.262	0.049	−0.2	0.283	0.072	−0.0005	0.5	0.003	0.02	0	—	—
M4	0.328	0.061	−0.2	0.283	0.091	−0.0005	0.5	0.003	0.02	0	—	—
ADD	0.273	0.049	−0.2	0.5	0.06	−0.0001	0.5	0.003	0.5	0.0009	0.05	0.5
REC	0.273	0.049	−0.2	0	0.06	−0.0001	0	0.003	0	0.0009	0.05	0
NEU	0.386	0	—	—	0	—	—	0	—	0	—	—

*U*, *s*, and *h* are the haploid genomic mutation rate, the effect or average effect of mutations in homozygotes, and the dominance coefficient or its average, respectively, for neutral (subscript neu), deleterious (del), slightly deleterious (sdel), lethal (let), and adaptive (adap) mutations. Model M1S was chosen to further include overdominant mutations as explained in the main text.

^a^For simulations with ancestral *N_e_* = 10^5^, the same parameters were used except that *s_sdel_* = −0.00001.

Partially recessive deleterious mutations in genic regions were assumed to follow a main mutational model shown to explain the changes in inbreeding load occurred in the laboratory population (Pérez-Pereira et al. 2021), which justifies its application in the present study. Absolute values for the mutational selection coefficients were obtained from a gamma distribution with shape parameter *β* = 0.33 and were assigned negative sign. The mean effect of mutations was $\bar{s}$ = −0.2. The dominance coefficient *h* of mutations was obtained from a uniform distribution between 0 and *e^ks^* (Caballero and Keightley 1994) when *s* ≥ −0.42, where *k* is a constant to obtain a mean dominance coefficient of $\bar{h}$ = 0.283. For values of *s* < −0.42, which would give values of *h* < 0.04 under the above model, *h* was obtained from a uniform distribution between 0 and 0.04. The above simulated mutational model itself generates lethal mutations at a rate 0.002 per haploid genome and generation, as sampled *s* values lower than −1 were assigned a value of *s* = −1. However, additional lethal mutations with *s* = −1 and *h* = 0.02 were also added at a rate 0.003 per haploid genome and generation. The joint distribution of *s* and *h* values of partially recessive and lethal mutations considered in the simulations is shown in Supplementary Fig. 1.

The haploid deleterious mutation rate resulting from the model (*U_del_* ≈ 0.05) and the average selection coefficient ($\bar{s}$ = −0.2) are concordant with the estimates obtained from mutation–accumulation experiments for a range of higher eukaryotic species reviewed by Halligan and Keightley (2009), with a median mutation rate of 0.04, and a mean of deleterious homozygous effect on fitness traits of −0.22. The assumed average dominance coefficient is supported by the range of estimates between 0.2 and 0.3 obtained in different studies (Crow and Simmons 1983; García-Dorado and Caballero 2000; Caballero 2006, 2020, p. 158; Agrawal and Whitlock 2011; Manna et al. 2011), and the distribution of *h* values assumed implies lower values for strongly deleterious mutations than for milder ones, as repeatedly observed (Mukai et al. 1972; Simmons and Crow 1977; Caballero 2006; Agrawal and Whitlock 2011; Huber et al. 2018).

Because the above deleterious mutational parameters are based on mutation–accumulation experiments, which are unable to detect deleterious mutations of very small effect (say, *s* > −5 × 10^−4^; García-Dorado et al. 2004), slightly deleterious additive mutations of effect *s_sdel_* = −0.0001 (for simulations with ancestral *N* = 10^4^ individuals) and *s_sdel_* = −0.00001 (for simulations with ancestral *N* = 10^5^ individuals) were also added in genic regions at a rate of *U_sdel_* = 0.05 mutations per haploid genome and generation. This type of mutations was also added in nongenic regions at a rate *U_sdel_* = 0.0125 (5% of all mutations in these regions).

Considering as a reference the model M1 described above, we now explain the other models of Table 1. The model M1S is identical to M1 but includes also advantageous (from now on adaptive) alleles in order to incorporate the possibility of adaptation to captivity (Orozco-terWengel et al. 2012). Because the foundation of the laboratory population from the wild was 200 generations in the past, we then assumed that, after arrival to the lab, a proportion 0.003 of neutral mutations became advantageous for adaptation to lab conditions. This implies a haploid genome rate of adaptive mutations of about *U_adap_* = 0.0009 per generation. The proportion 0.003 is obtained from the observed proportion of SNPs assumed to be under adaptation to captivity by Orozco-terWengel et al. (2012). We assumed that these adaptive mutations had additive gene action and a selection coefficient of *s* = 0.05, which is the largest estimated effect observed by Sattath et al. (2011).

We also considered alternative mutational models (see Table 1) involving a larger effect of slightly deleterious mutations (*s_sdel_* = −0.0005) (models M2 and M2S), also a higher rate of slightly deleterious mutations (model M3), and an additional higher rate of deleterious mutations (model M4). Models were also considered where all types of mutations were additive (ADD) or fully recessive (REC). Finally, a model was considered were all mutations were neutral (NEU). The results of the above simulation models were contrasted with those obtained empirically in order to ascertain which of them explains better the observed data.

Overdominant mutations were obtained by assuming a positive selection coefficient (*s* > 0) and *h* = 1.5 and were assigned only to genic regions. Selection coefficients were obtained from a gamma distribution with shape parameter *β* = 0.66 and mean effect $\bar{s}$ = 0.026. These parameters were obtained by fitting a gamma distribution to the empirical selection coefficients of overdominant mutations with values lower than 0.1 reported by Thurman and Barrett (2016) (Supplementary Fig. 2). We considered models including only deleterious mutations (M1, M2, M3, M4), others including also adaptive mutations (M1S, M2S, ADD, REC), and one model (M1S) where overdominant mutations were also added at increasingly larger rates from *U_o_* = 0.5 × 10^−7^ to 8 × 10^−7^ per haploid genome and generation. These rates generated a total number of genomic overdominant segregating loci in the last generation ranging from about 3 to almost 50. For simulations with ancestral *N* = 10^5^, the rate of overdominant mutations was reduced by a factor of 10 in order to obtain the same approximate number of genomic overdominant segregating loci as for the simulations with *N* = 10^4^. Ten independent simulations of each of the above scenarios were run for each of the 4 chromosome arms separately in the case of simulations with *N* = 10^4^. Three replicates were run instead in the case of simulations with *N* = 10^5^, given the high computer load. The mean numbers of segregating neutral (*n_neu_*), slightly deleterious (*n_sdel_*), deleterious (*n_del_*), lethal (*n_let_*), adaptive (*n_adap_*), and overdominant (*n_o_*) mutations at the last simulated generation were counted, and their mean frequencies (*q*) were calculated.

Additive variance (*V_A_*), dominance variance (*V_D_*), and inbreeding load (*B*; Morton et al. 1956) were calculated as *V_A_* = Σ2*α*^2^*pq*, *V_D_* = Σ(2*dpq*)^2^, and *B* = Σ2*dpq*, where the summation is for all segregating loci; *p* and *q* are the frequencies of the wild-type and mutant alleles, respectively; *α* is the average effect of an allelic substitution; and *d* is the difference between the heterozygote and the mid homozygous values. For slightly deleterious, deleterious, and lethal mutations, *d* = *s*(*h*–1/2), and *α* = *sh*–2*dq* (see, e.g. Caballero 2020, p. 180). For overdominant loci, the simulated model (1, 1 + *sh*, 1 + *s*) was scaled to the classical one (1–*s_A_*, 1, 1–*s_a_*) by dividing the genotype fitness values by that of the heterozygote, so that *s_A_* = *sh*/(1 + *sh*) and *s_a_* = *s*(1 + *h*)/(1 + *sh*), *d =* (*s_A_* + *s_a_*)/2 and *α = qs_a_–ps_A_*. The fractions of *V*_A_, *V*_D_, and *B* attributable to segregating overdominant loci were also obtained as for the other types of loci. The total values of *V*_A_, *V*_D_, and *B* were obtained as the sum of values from all types of segregating selective mutations. As for the empirical data, the linkage disequilibrium statistic *r*^2^ between consecutive neutral SNPs was computed using PLINK (Purcell et al. 2007). Nucleotide diversities (*π*) were obtained with VCFtools (Danecek et al. 2011) to calculate each 100-kb window average considering only neutral SNPs. To allow comparisons with the empirical values, each mean value of *π* was scaled by the ratio between the total number of SNPs per arm in the empirical data and in the simulations. The results of simulations from the 4 chromosome arms for each overdominant mutation rate (*U_o_*) were averaged, so that 10 replicates considering the complete autosomal genome were available for obtaining confidence intervals of estimates.

Because the different forms of selection (background selection, selective sweeps, and balancing selection) have an impact on neutral genetic variation, particularly in regions of low recombination, we focused on the relationship between genomic diversity and recombination frequency. Thus, we examined the relationship between the mean nucleotide diversity (*π*) and the mean recombination rate (*c*) of the 100-kb windows for both the empirical and simulated data, combining the 4 chromosome arms. The relationship between *π* and *c* values of all genomic windows for empirical and simulated results was fitted using a rational function of the type *π* = [(*a* × *c*) + *b*]/(*c* + *d*), where *a* is the asymptote of the fitted curve and *b*/*d* is the intercept in the ordinate. This was done in R as follows: model <-nls(pi_vec∼(*a* * c_vec + *b*)/(c_vec + *d*), start = list(*a* = 0.006, *b* = 0.002, *d* = 1)), where pi_vec is the vector of *π* values and c_vec is the vector of *c* values for the genomic windows. For the simulation results, the curve was fitted to the average values over simulation replicates. We compared the predicted curves of the different models with the empirical curve by calculating their RMSE and also by an AIC test, also using R. We also contrasted the average nucleotide diversity of the entire autosomal genome (*π*) of empirical and simulated data as well as the average nucleotide diversity in genomic regions with recombination rate lower than 0.25 centimorgans (cM) per megabase (Mb) (*π_c <_*  _0.25_) using a Kolmogorov–Smirnov (K–S) test. Ninety-five percent confidence intervals for simulation results were also calculated to contrast simulation and empirical results. Confidence limits for *π* and linkage disequilibrium (*r*^2^) values for empirical data were obtained by bootstrapping across window values, with 10,000 samplings of windows with replacement using a custom C program.

## Results

### Estimation of effective population size across generations

**Fig. 1.**
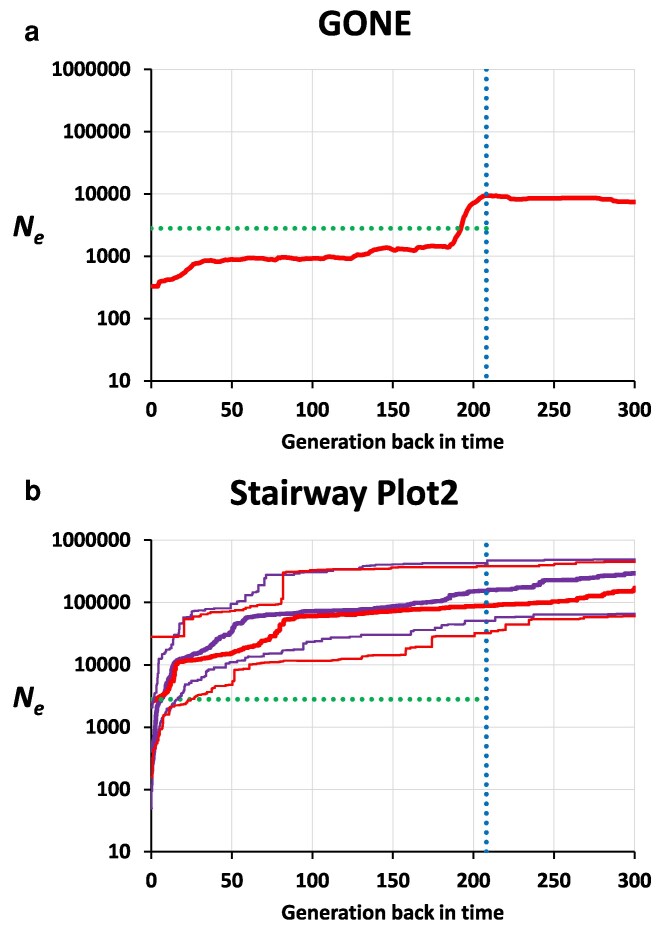
Estimates of historical effective population size (*N_e_*) obtained from linkage disequilibrium between SNPs (software GONE, panel a), or from the SFS of variants (software Stairway Plot2, panel b). For this latter, the thick lines are median values and the thin dotted lines are the 95% confidence limits, with purple lines denoting the results using the silent sites and red lines those using the synonymous sites. The vertical blue-dotted line indicates the time when the population was captured and maintained in the laboratory since then. The horizontal green-dotted line indicates the total number of individuals maintained in the laboratory population.

The results from GONE, shown in Fig. 1a, indicate that the ancestral population (the wild population of origin) had an approximate *N_e_* of 10,000 individuals, which suddenly dropped to about 1,000 individuals at the time of capture (208 generations back, shown by the blue vertical dotted line), which then was held approximately constant over the whole period of 208 generations, except by a drop in the last 25 generations. This final drop is an expected artifact due to population structure (Santiago et al. 2020; Novo et al. 2023a) because the population was not fully panmictic but maintained in bottles with circular mixing. Computer simulations confirmed this effect (Supplementary Fig. 3). A value of *N_e_* ≈ 1,000 individuals in relation to a total number of *N* ≈ 2,800 individuals (horizontal green-dotted line) implies a ratio *N_e_*/*N* = 0.36, which is in agreement with typical estimates of captive *Drosophila* populations (Frankham 2007).

The estimates using the software Stairway Plot2 (Fig. 1b) show an ancestral *N_e_* higher than that obtained by GONE (of the order of 300,000 individuals) and a continuous decline over the last 300 generations and particularly in the last 50, down to values below 1,000 (silent sites) or about 5,000 (synonymous sites) individuals. Estimates from around 20 generations ago (about 1 yr back) were of the order of 10,000 individuals, and estimates around 100 generations back (about 5 yr back) were of the order of 70,000 individuals, which is clearly incorrect, as the entire population was maintained at a total of about 2,800 individuals for a period of 208 generations. Thus, the estimations from Stairway Plot2 must be considered overestimations, at least in the most recent period.

Alternative indirect estimates of ancestral *N_e_* can also be obtained from the genetic diversity observed in the sample. The nucleotide diversity (*π*) per nucleotide in silent positions was estimated from the fSFS as 0.0036 (excluding 2L). Considering that *θ* = 4*N_e_u*, a rough average estimation may be obtained as *N_e_* = *θ*/(4*u*), i.e. *N_e_*(*π*) = 0.0036/(4 × 3e−9) = 300,000, in agreement with the ancestral estimate of *N_e_* from Stairway Plot2.

### Impact of overdominance on nucleotide diversity

**Fig. 2.**
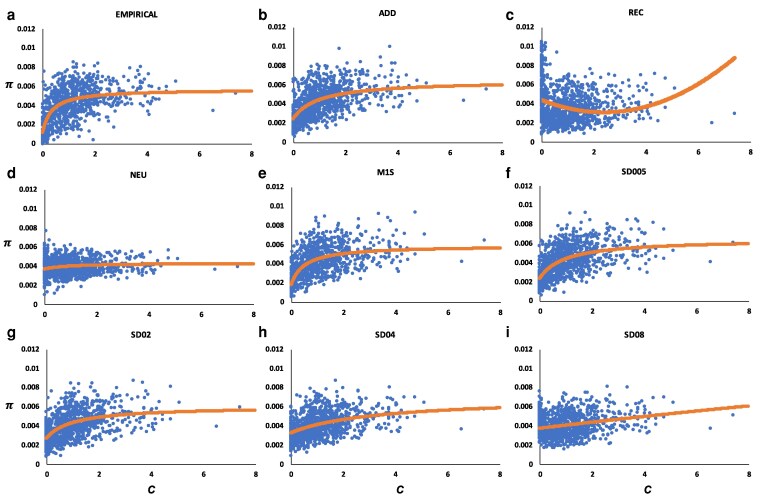
Relationship between the mean nucleotide diversity (*π*) and the mean recombination rate (*c*) in centimorgans (cM) per megabase (Mb) for 100-kb genomic windows considering the 4 autosomal chromosome arms, for simulation that assume an ancestral effective population size of *N_e_* = 10^4^. The curve shown in each graph represents the rational function fitted to the data (except for the REC model for which this function cannot be fitted and a polynomial curve is presented instead). a) Empirical data. (b–i) Simulation results without (panels b–e) or with (panels f–i) overdominant mutations. ADD, REC: all mutations are assumed to be additive or fully recessive, respectively. NEU: only neutral mutations are assumed. M1S: mutations assuming the parameters shown in Table 1. SD005-SD08: mutations assuming the same parameters as for model M1S and also including overdominant mutations at a rate (*U_o_*), 0.5 × 10^−7^ (SD005), 2 × 10^−7^ (SD02), 4 × 10^−7^ (SD04), 8 × 10^−7^ (SD08). Simulation data is based on the average of 10 replicates.

**Fig. 3.**
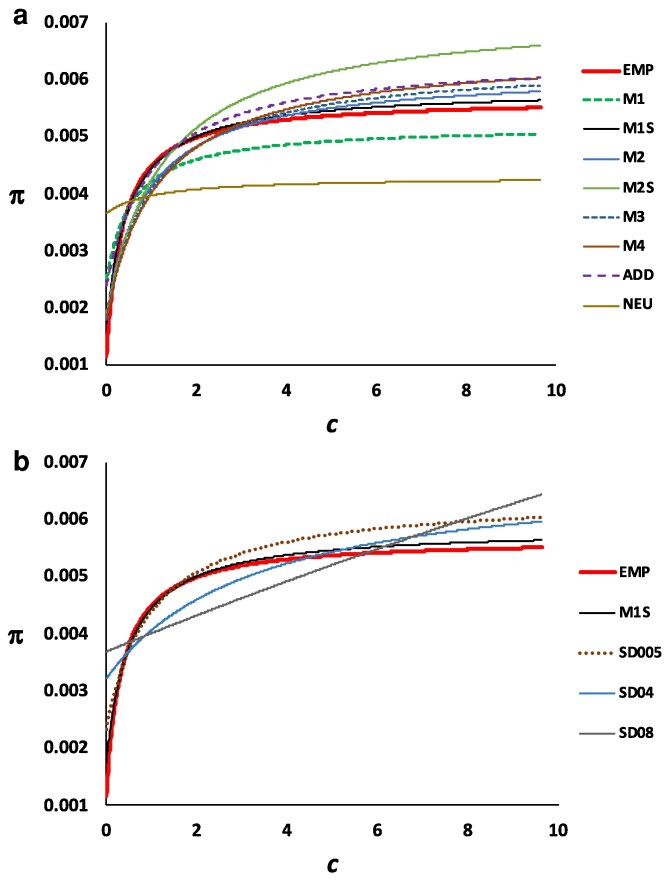
Rational function fitted to the mean values of nucleotide diversity (π) with respect to the mean recombination rate (*c*) in centimorgans (cM) per megabase (Mb) for 100-kb genomic windows considering the 4 autosomal chromosome arms, for simulation that assume an ancestral effective population size of *N_e_* = 10^4^. a) Empirical (EMP) data and models excluding overdominant mutations, as shown in Table 1. b) Empirical (EMP) data, model M1S excluding overdominant mutations, and models assuming the same parameters as for model M1S, and also including overdominant mutations at a rate (*U_o_*), 0.5 × 10^−7^ (SD005), 4 × 10^−7^ (SD04), 8 × 10^−7^ (SD08).

Simulations initially assumed the demographic history inferred by GONE estimations (Fig. 1a), with an ancestral *N_e_* = 10^4^ and a recent one of *N_e_* = 10^3^. Figure 2 shows the relationship between the mean nucleotide diversity for each 100-kb window (*π*) and the mean recombination rate (*c*) of that window for the empirical data (Fig. 2a) and some of the different simulated models presented in Table 1 and for models with increasing rates of overdominant mutations. The figures also show the rational function fitted to the data (for the recessive model—panel C—the function could not be fitted because of its inverted shape, and a polynomial regression is shown instead). These functions are also shown in Fig. 3 for a better comparison of the different models.

For the empirical data, the lower the recombination rate, the lower the nucleotide diversity, as expected under purifying selection, resulting in an increase of the function as the recombination rate (*c*) increases, to reach an asymptotic plateau for high *c* values. First, we compared the empirical data with the models assuming no overdominant mutations. A similar function shape was obtained with all the different models (Fig. 3a) except with the neutral (NEU) model (Fig. 2d and Fig. 3a) and the fully recessive (REC) model (Fig. 2c). A comparison of the fit between the function for different models and that from the empirical data is shown in the upper part of Table 2. The lowest RMSE corresponds to the model M1S and the largest to the neutral model. Accordingly, the lowest and highest AIC values correspond to these same models. The K–S test comparing the *π* values between the empirical and simulation models for all genomic windows and for those windows with recombination rate *c* < 0.25 (Table 2) also shows that the highest *P*-value is obtained for the M1S model. Therefore, the model M1S, which included adaptive mutations, is the closest to the empirical data among the different models investigated. We thus considered this as the base model, to which overdominant mutations were added. Some of the models with overdominance are presented in Fig. 2 (panels f–i) for increasing rates of overdominant mutations. The middle part of Table 2 compares the model M1S and the overdominant models with the empirical data. All results suggest that the M1S model explains better the empirical data than any model assuming overdominant mutations, the second column showing the average number of overdominant mutations segregating in the genome for each of the models. The comparison between the rational function fitted to the empirical data, the simulated data with model M1S (no overdominance), and some of the overdominant models (M1S + overdominant mutations) is shown in Fig. 3b. Overdominance clearly increases the intercept of the function with the ordinate (see also Fig. 2) by increasing the *π* values of windows with low recombination rate.

**Table 2.** Statistical comparison between the results obtained with the different models simulated and the empirical data.

	*n_o_*	RMSE	AIC	K–S (*π*)	K–S (*π_c <_* _0.25_)
**No OD models—ancestral *N_e_* = 10^4^**					
M1	0	0.000432	−14,931.65	1.91E−07	0.00E + 00
**M1S**	0	**0.000099**	**−17,774.54**	**1.91E**−**02**	**1.04E**−**06**
M2	0	0.000215	−16,280.39	2.18E−05	4.10E−12
M2S	0	0.000744	−13,885.35	1.12E−03	1.13E−08
M3	0	0.000285	−15,736.76	5.15E−07	6.16E−09
M4	0	0.000359	−15,291.43	8.47E−07	3.38E−11
ADD	0	0.000367	−15,246.65	4.53E−04	4.56E−13
REC	0	—	—	7.93E−13	0.00E + 00
NEU	0	0.001112	−13,109.60	0.00E + 00	0.00E + 00
**No OD vs OD models—ancestral *N_e_* = 10^4^**					
**M1S**	**0**	**0.000099**	**−17,774.54**	**1.91E**−**02**	**1.04E**−**06**
SD005	2.8	0.000368	−15,244.14	1.42E−04	3.38E−11
SD01	5.1	0.000179	−16,634.28	1.76E−06	0.00E + 00
SD02	10.8	0.000172	−16,712.18	1.08E−06	0.00E + 00
SD04	22.3	0.000344	−15,368.97	1.68E−09	0.00E + 00
SD06	36.0	0.000493	−14,676.43	2.21E−12	0.00E + 00
SD08	47.3	0.000549	−14,470.24	1.97E−13	0.00E + 00
**No OD vs OD models—ancestral *N_e_* = 10^5^**					
**M1S**	**0**	**0.000207**	**−16,363.90**	**1.86E**−**07**	**1.17E**−**07**
SD005	2.67	0.000399	−15,703.85	2.51E−02	8.97E−24
SD01	5.33	0.000292	−15,103.53	5.13E−05	1.41E−19
SD04	20.5	0.000569	−14,416.83	7.85E−04	4.65E−14

The upper part of the table compares models assuming no overdominant (OD) mutations (see Table 1) assuming an ancestral *N_e_* = 10^4^ individuals, while the middle part compares the best model found without overdominant mutations (M1S, in bold) with models assuming different OD mutation rates (U_o_): 0.5 × 10^−7^ (SD005), 1 × 10^−7^ (SD01), 2 × 10^−7^ (SD02), 4 × 10^−7^ (SD04), 6 × 10^−7^ (SD06), 8 × 10^−7^ (SD08). The lower part of the table gives results assuming an ancestral *N_e_* = 10^5^ individuals. The second column (*n_o_*) shows the average number of segregating OD mutations in the analyzed simulated populations. RMSE and AIC are the root mean squared error and Akaike information criterion, respectively, comparing the rational function fitted to the empirical π values from genomic windows with those fitted to simulated data from different models. K–S refers to the probability value of the Kolmogorov–Smirnov test comparing the empirical π values from genomic windows with those simulated data from different models.

**Fig. 4.**
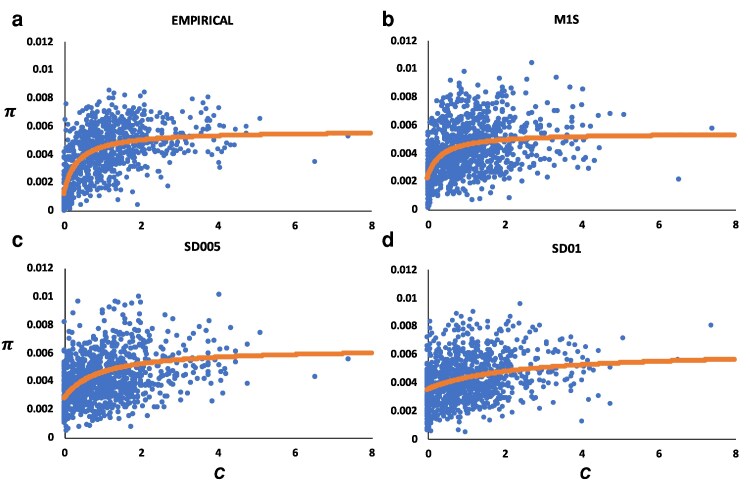
Relationship between the mean nucleotide diversity (*π*) and the mean recombination rate (*c*) in centimorgans (cM) per megabase (Mb) for 100-kb genomic windows considering the 4 autosomal chromosome arms, for simulation which assume an ancestral effective population size of *N_e_* = 10^5^. The curve shown in each graph represents the rational function fitted to the data. a) Empirical data. b) Simulation results for model M1S without overdominant mutations. (c and d) Models assuming the same parameters as for model M1S and also including overdominant mutations at a rate (*U_o_*), 0.05 × 10^−7^ (SD005) and 0.1 × 10^−7^ (SD01). Simulation data is based on the average of 3 replicates.

Results assuming an ancestral population size of 10^5^ individuals rather than of 10^4^ individuals confirmed the previous results. Figure 4 shows the relationship between the mean nucleotide diversity and the mean recombination rate, and the rational functions fitted, analogous to Fig. 2, for simulation models assuming or not overdominance. The corresponding statistical tests are shown in the lowest part of Table 2. The model assuming no overdominance fits generally better to the empirical data than the models assuming overdominance, supporting the previous results with an ancestral population size of 10^4^ individuals. Interestingly, the fit to empirical data of simulations assuming population size 10^5^ was slightly worse than that assuming population size 10^4^, as suggested by the higher RMSE obtained with size 10^5^ than with size 10^4^ for the 4 comparable cases (Table 2). Thus, in what follows, we focus on the results assuming an ancestral population size of 10^4^ individuals.

**Fig. 5.**
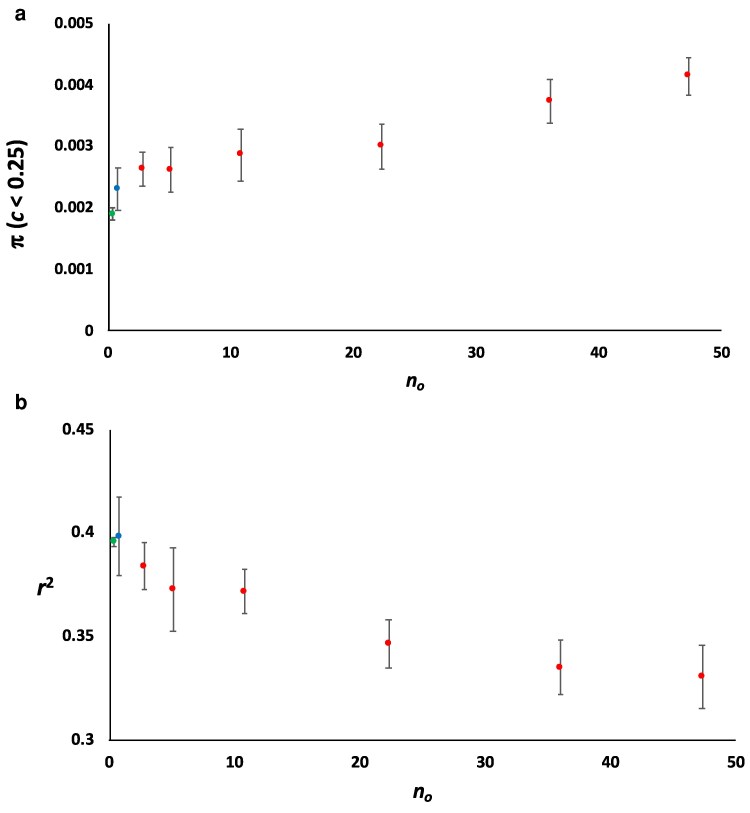
Average nucleotide diversity (*π*; panel a) and linkage disequilibrium between consecutive loci (*r*^2^; panel b) for a range of simulated overdominant mutation rates, ranging from *U_o_* = 0.5 to 8 × 10^−7^, with the *x*-axis indicating the mean number of overdominant segregating mutations (*n_o_*) for each overdominance mutation rate. Results refer to simulations that assume an ancestral effective population size of *N_e_* = 10^4^. The values of *π* refer to 100-kb genomic windows with average recombination rate *c* < 0.25 cM/Mb. Dots indicate the mean *π* or *r*^2^ based on the 10 simulated replicates, and bars indicate the 95% confidence intervals. The green dots (first from the left-hand side) refer to the empirical data with confidence limits obtained by bootstrapping, the blue dots (second from the left-hand side) correspond to the simulation results assuming the mutation model M1S without overdominance, which parameters are shown in Table 1. The remaining red dots refer to the models assuming the same parameters as for model M1S but also including overdominant mutations at a rate (*U_o_*) ranging from 0.5 × 10^−7^ to 8 × 10^−7^.

The average values of *π* for windows with *c* < 0.25 and *r*^2^ for the empirical data (green dots), for the model M1S (no overdominance; blue dots), and for the models with overdominance (red dots) are shown in Fig. 5. While the confidence limits of M1S and the empirical data overlap in both figures, only that for the lowest overdominance model does so for *r*^2^. The effect of overdominance is an increase in *π* for low recombination rates and a decrease in the linkage disequilibrium between consecutive neutral variants. In summary, none of the models with overdominant mutations, except perhaps that with a very low number of overdominant mutations in terms of linkage, were compatible with the empirical results.

### Impact of overdominance on allele frequencies, genetic variance, and inbreeding load

**Fig. 6.**
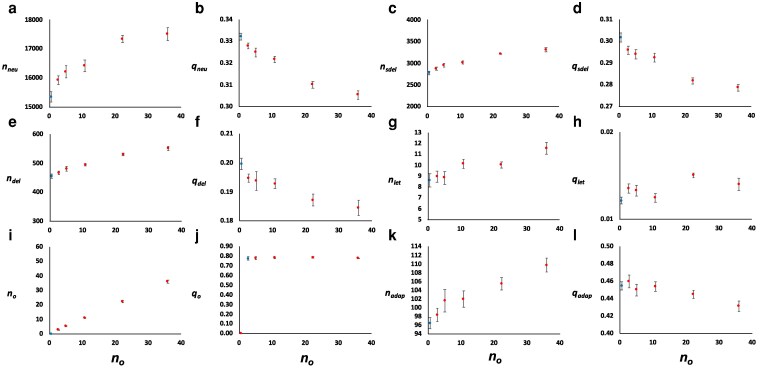
Mean number (*n*) and average frequency (*q*) of neutral (subscript *neu*, panels a, b), partially recessive deleterious (*del*, panels e, f), additive slightly deleterious (*sdel*, panels c, d), lethal (*let*, panels g, h), adaptive (*adap*, panels k, l), and overdominant (*o*, panels i, j) mutations. Results refer to simulations that assume an ancestral effective population size of *N_e_* = 10^4^. The blue dots (first from left-hand side in each panel) correspond to the simulation results assuming the mutation model M1S without overdominance, which parameters are shown in Table 1. The red dots refer to the models assuming the same parameters as for model M1S but also including overdominant mutations at a rate (*U_o_*) ranging from 0.5 × 10^−7^ to 8 × 10^−7^, with the *x*-axis indicating the mean number of overdominant segregating mutations (*n_o_*) for each overdominance mutation rate. Results are based on 10 simulation replicates and bars indicate 95% confidence intervals.

Figure 6 depicts the mean number (*n*) and frequency (*q*) of segregating neutral (*neu*), slightly deleterious (*sdel*), deleterious (*del*), lethal (*let*), adaptive (*adap*), and overdominant (*o*) mutations with respect to the mean number of overdominant segregating mutations (*n_o_*) corresponding to each simulated overdominant mutation rate. Increasing overdominance led to an increase in the number of neutral and deleterious segregating mutations and a slight decrease in their average frequency. The average frequency of overdominant mutations (*n_o_*) was about 0.75, as expected from the asymmetrical model of overdominance considered.

**Fig. 7.**
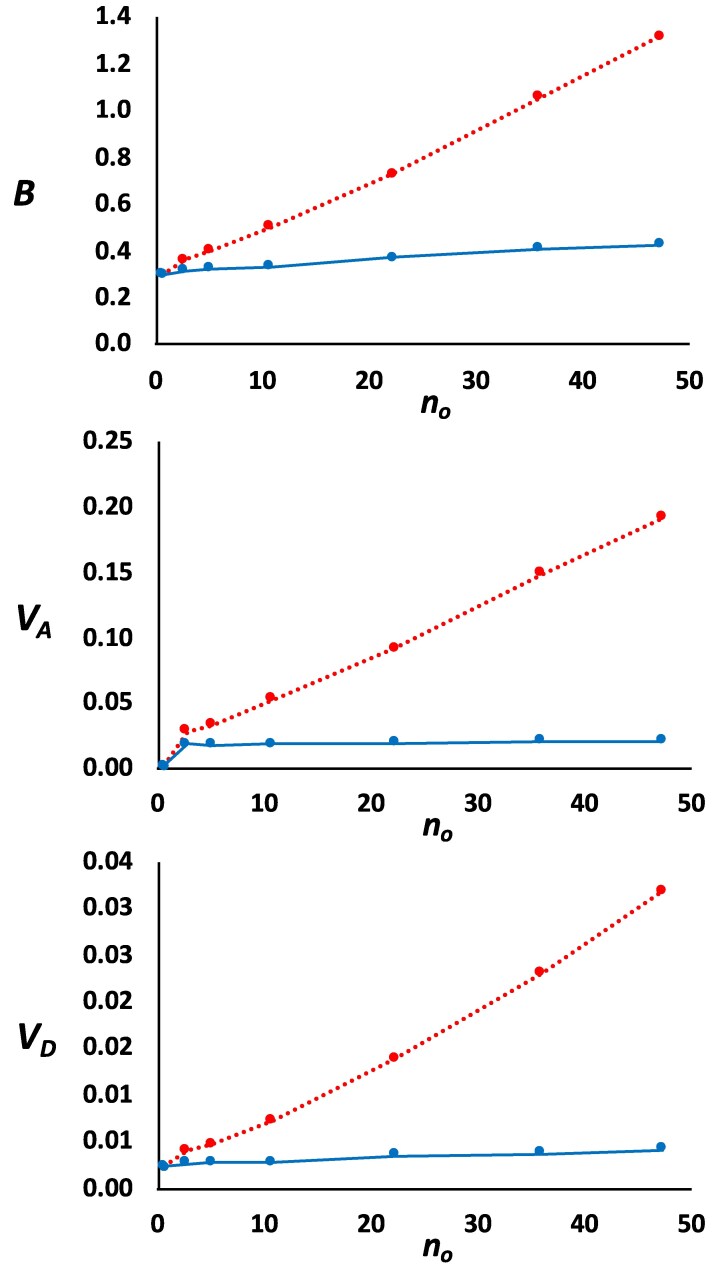
Changes of inbreeding load (*B*), additive variance (*V*_A_) and dominance variance (*V*_D_) for models assuming the same parameters as for model M1S (parameter in Table 1), but also including overdominant mutations occurring at different rate *U_o_*, leading to different numbers of segregating overdominant mutations (*n*_o_). Results refer to simulations that assume an ancestral effective population size of *N_e_* = 10^4^. Red dots and dotted lines give the overall values contributed for all kind of loci, with the *x*-axis indicating the mean number of overdominant segregating mutations (*n_o_*) for each overdominance mutation rate. Blue dots and continuous lines indicate the contribution from segregating nonoverdominant loci. Results are based on 10 simulation replicates.

Finally, Fig. 7 shows the changes in the inbreeding load (*B*), additive variance (*V_A_*), and dominance variance (*V_D_*), as the average number of segregating overdominant mutations (*n_o_*) increased. The values of *B*, *V_A_*, and *V_D_* arising from nonoverdominant mutations (blue lines) were kept almost invariable regardless of the level of overdominance. As expected, the values of *B*, *V_A_*, and *V_D_* when all types of mutations were considered (red lines) increased substantially with increasing overdominance due to the input of these loci.

## Discussion

We have attempted to quantify the contribution of overdominant loci to genetic variation by using a combined approach of genomic analysis of empirical data and computer simulations. The objective was not to ascertain the precise genetic model explaining the variation in the population but a model which provides a scenario as close as possible to that occurred in the experimental population, in order to investigate the impact of adding overdominant variation. By quantifying the relationship between the magnitude of nucleotide diversity across genomic regions with different recombination rates, our results show that a parsimonious model of background selection from deleterious mutations and adaptation to captivity explains the observed data better than models including overdominant loci.

A key circumstance which allows the comparison between simulation and empirical results is that the recent demography of the population is known, as the population was kept in the laboratory for more than 200 generations with an approximately constant size of 2,800 individuals. We applied the software GONE to obtain estimates of *N_e_* during this period and further back with consistent results showing a constant *N_e_* of about 1,000 individuals (Fig. 1a) in agreement with previous estimates using a lower set of individuals (Novo et al. 2023b). GONE *N_e_* estimates also indicate an ancestral *N_e_* of about 10,000 individuals, which suddenly declines to *N_e_* = 1,000 almost at the precise time of capture (Fig. 1a). A new version of the software recently released (GONE2; Santiago et al. 2025) could not be applied in this case, as this version is focused on a maximum of 150 generations back in time, shorter than the time period involved in our experiment.

The estimate of ancestral *N_e_* with GONE contrasts with those obtained from the site frequency spectrum (SFS; Stairway Plot2) or from indirect measures from the observed genetic diversity (*θ*), which suggest values around 300,000 individuals. The estimates from GONE are based on linkage disequilibrium between SNPs and are more reliable for recent than for ancient estimates. In contrast, estimates from the SFS are more reliable for ancient than for recent estimates, and estimates from *θ* refer to the harmonic mean of estimates of *N_e_* across the whole history of coalescent events in the species. Our main simulation results correspond to a model where the demographic history of the population is that inferred by GONE, with an ancestral *N_e_* of 10^4^ individuals, but we carried out additional simulations assuming an ancestral *N_e_* of 10^5^ individuals, and the results obtained confirmed those with the lower *N_e_* (Fig. 4 and Table 2). The model excluding overdominant mutations fitted better to the empirical results than the models including overdominance, even at a very low number of overdominant loci (Table 2). Interestingly, the results assuming an ancestral *N_e_* = 10^4^ fitted better (lower RMSE) than those assuming *N_e_* = 10^5^. Thus, even when assuming a large ancestral *N_e_* consistent with diversity-based estimates, our analyses support the conclusion that widespread segregating overdominant loci are unlikely in this system.

The average empirical mean value of nucleotide diversity at the sampling time was 0.0036 (0.0033 excluding 2L). Because our experimental population was maintained for about 200 generations with an effective size of about 1,000 individuals, the neutral diversity should have been about 10% higher at the time of the population foundation, i.e. around 0.0040, which is within the lowest range of those found in European populations (median around 0.0048) by the meta-analysis of Kapun et al. (2021). Our simulations assuming the absence of overdominance (model M1S) provided a value of 0.0040 ± 0.0001 at the time of sampling, very close to the empirical one. More importantly, the average nucleotide diversity observed in regions of low recombination (*c* < 0.25 cM/Mb) was very close for empirical (0.0019) and simulation data in the absence of overdominant mutations (0.0023 ± 0.0003). The average linkage disequilibrium between consecutive neutral SNPs (*r*^2^ = 0.396) was also very similar between the empirical and simulated datasets without overdominance (*r*^2^ = 0.398), but as soon as overdominance was added, the linkage disequilibrium decreased accordingly away from the empirical value. Finally, the simulated inbreeding load obtained in the absence of overdominance (model M1S) was about 0.32, which is somewhat higher than that estimated at generation 201 by Pérez-Pereira et al. (2021) (0.151 ± 0.070) comparing the means of pupae productivity of non-inbred and inbred (full-sib progeny) flies. This difference may not be surprising because pupae productivity is not likely to encompass all components of global fitness, as intended in our simulations.

Although our results suggest that a parsimonious model of background selection and adaptation to captivity is able to explain the empirical results and that none of the models of overdominance are compatible with the empirical data, a minor contribution from overdominant loci cannot be fully discarded given the uncertainty in the simulation parameters. The lowest overdominance mutation rates considered in our simulations (*U_o_* = 0.5 × 10^−7^, 1 × 10^−7^, and 2 × 10^−7^) would involve only about *n_o_* = 3, 5, and 11 overdominant simulated loci segregating in the population, respectively. The proportion of these mutations in relation with the deleterious and lethal ones was about 0.6%, 1%, and 2%, respectively. This number of overdominant loci may be, in fact, conservatively large, as we assumed a model of overdominance with effects within the lowest range found by Thurman and Barrett (2016) (Supplementary Fig. 2a). In their meta-analysis, they found 70 out of the 3,416 (or 2%) selection coefficients examined that could be assigned to overdominant selection. However, these authors already warned about the overinterpretation of their results because of statistical biases, so that the estimates would be expected to have a large error. Thus, we conservatively considered only those effects lower than 0.1 which had a gamma-shaped distribution with mean effect 0.026 (Supplementary Fig. 2b). Assuming these numbers of overdominant mutations (*n_o_* = 3, 5 and 11), our simulation results suggest that they would be responsible for about 13%, 18%, and 34% of the inbreeding load, respectively (Fig. 7a). The impact of these overdominant mutations would also be an increase in both the additive and dominance variances, of about a 35%, 46%, and 54%, respectively (Fig. 7b and c). Thus, a relatively small number of overdominant loci would account for substantial amounts of inbreeding load and genetic variation.

Pseudo-overdominance, the result of interactions between deleterious recessive mutations at closely linked loci in repulsion phase (Carr and Dudash 2003; Charlesworth and Willis 2009; Draghi and Whitlock 2015), can mistakenly appear as heterozygote advantage, and it is seen as a mechanism maintaining neutral diversity, especially in low recombination regions and small populations (Schou et al. 2017; Becher et al. 2020; Gilbert et al. 2020). Thus, pseudo-overdominance may contribute to an increased nucleotide diversity observed in genomic regions of low recombination (Abu-Awad and Waller 2023). However, this is accounted for in our simulations. It has been theoretically shown that partially recessive deleterious mutations can result in pseudo-overdominance when 2*N_e_s* < 2.5 if linkage is sufficiently tight (Zhao and Charlesworth 2016). For our simulated effective population size (*N_e_* ≈ 1,000), this implies mutations with *s* < −0.00125. In our simulation model, the proportion of mutations with this effect and partially recessive gene action account for about 15% of all deleterious mutations. The simulation model including exclusively additive mutations (ADD) would be devoid of pseudo-overdominance. This model was clearly less appropriate than the partially recessive model (M1S) to explain the empirical data (Table 2), suggesting that the contribution from pseudo-overdominance is necessary to explain the experimental results. However, a fully recessive model (REC), which would imply a large amount of pseudo-overdominance, is clearly inappropriate.

In our simulations, we considered a model of deleterious mutations found to be consistent with the changes in inbreeding load observed in the population (Pérez-Pereira et al. 2021) with some modifications. Although there are other possible models to be considered, this model is also shown to explain rather well the diversity observed in the population. This model, when excluding the slightly deleterious set of mutations, implies an average heterozygote effect (*sh*) of mutations amounting to −0.012, which is within the range of values (−0.01 to −0.02) suggested from quantitative genetic estimates for *Drosophila* (Crow and Simmons 1983; Charlesworth and Hughes 1999; Lynch et al. 1999; Charlesworth 2015). This model of mutations refers to a fraction of mutations of relatively large effect which can be detected in mutation–accumulation experiments (García-Dorado et al. 2004; Halligan and Keightley 2009) and which possibly includes not only single-nucleotide mutations or small indels but also transposable element insertions and larger indels with large effects on fitness (Charlesworth 2015). These relatively large-effect mutations are expected to be the most relevant ones when it comes to assessing the impact of inbreeding depression and the evolution of fitness in small populations and short periods of time (e.g. Caballero and Keightley 1998; Caballero et al. 2002; Pérez-Pereira et al. 2021). However, many deleterious mutations of smaller effect, as quantified in population genomic analyses, are not included in the model. Thus, we added a class of slightly deleterious mutations of small effects (*s* = −0.0001 or −0.00001) with additive gene action. This addition implied that the global average heterozygote effect (*sh*) of mutations got down to −0.006 or −0.0006, closer to the value estimated from population genomics analyses (around −0.001; Charlesworth 2015). The number of neutral and deleterious mutations increased for increasing levels of overdominance, which can be explained by the general increased diversity maintained by overdominance.

Our study has the unusual advantage that the demographic history of the population is known. This knowledge is very relevant because the impact of selection on genetic variation is well known to be highly dependent on population demography (García-Dorado 2012; Torres et al. 2019). However, we can point out several limitations of our study. First, we considered that deleterious and overdominant mutations mainly occurred in genic regions, which account for about 40% of the genome. In nongenic ones, we only considered slightly deleterious mutations. Although other deleterious and overdominant mutations might also occur in nongenic regions, we believe this approximation can be more realistic than assuming mutations with strong selective effects randomly scattered along the genome. In fact, Becher et al. (2020) fully dismissed mutation in intergenic regions in their simulations investigating the impact of associative overdominance due to partially recessive deleterious and advantageous mutations. Second, modeling advantageous mutations is hard, given the scarcity of mutational parameters for this type of mutations. We assumed a proportion of neutral mutations becoming adaptive from the observations of Orozco-terWengel et al. (2012) for adaptation to captivity and the maximum selective coefficient observed by Sattath et al. (2011). Nevertheless, our results suggest that the inclusion of these mutations improved the explanatory model (model M1S vs M1). Third, because our empirical data refer to a laboratory population, it may occur that the conditions generating balancing selection in nature may have been absent or attenuated under laboratory conditions. However, the population was maintained for a relatively short (evolutionary speaking) period of time in the lab (around 200 generations). Thus, overdominant alleles could have been maintained for that period (even if selection is relaxed), given the large population size and the high expected segregation frequency of overdominant alleles. Nevertheless, our results cannot be extrapolated to other natural or laboratory populations and cannot serve as a global rebuttal of overdominance variation in nature. Finally, our simulated model did not consider the possibility of chromosomal inversions segregating in the population. This would involve regions of restrained recombination, possibly subjected to strong pseudo-overdominance. However, under this latter scenario, the footprint of balancing selection would be exacerbated in the real data with respect to simulations, making our results more conservative. All in all, the remarkable agreement between the empirical and simulation results suggests that the above limitations have a restricted impact in the study.

In conclusion, our results support the idea that the most parsimonious model of background selection, due to partially recessive deleterious mutations and adaptation to captivity, can explain the genetic diversity observed in our experimental data better than models including overdominance. Of course, given the limitations of the models that can be simulated, a contribution from overdominance cannot be fully dismissed as a source of genetic variation and inbreeding depression, but its contribution should be, at most, minor.

## Supplementary Material

iyag050_Supplementary_Data

## Data Availability

Simulation codes and scripts are available at Github address https://github.com/armando-caballero/Overdominance-in-Drosophila. FASTQ files have been deposited in the NCBI Sequence Read Archive (SRA) database under accession numbers SAMN55326084—SAMN55326134 (BioProject accession: PRJNA1422961). Supplemental material available at GENETICS online.
