# Supplementary material for: Overdominance for fitness: a genomic comparison between empirical and simulated data with Drosophila melanogaster
Source: Genetics. 2026 Feb 24;233(1):iyag050. doi: 10.1093/genetics/iyag050 (PMC13147538; doi:10.1093/genetics/iyag050)
Supplement: iyag050_Supplementary_Data [file iyag050_supplementary_data.docx]

**Supplemental Material**

**Overdominance for fitness: A genomic comparison between empirical and simulated data with *Drosophila melanogaster***

Inés González-Castellano, Humberto Quesada, Sebastián Ramon-Onsins, Aurora García-Dorado, Armando Caballero

**Joint distribution of selection (*s*) and dominance (*h*) coefficients used in the simulations**

Figure S1 shows the joint distribution of selection (*s*) and dominance (*h*) coefficients of partially recessive deleterious mutations used in the simulations.

**Figure S1.** 3D surface plot of the distribution of absolute frequencies for

combinations of selection coefficients (*s*) and dominance coefficients (*h*) of partially

deleterious mutations. See interactive HTML plot at Github repository:

<https://github.com/ines-gonzalezcastellano/overdominance-in-Drosophila>

**Empirical distribution of overdominant mutation effects compiled by Thurman and Barrett (2016)**


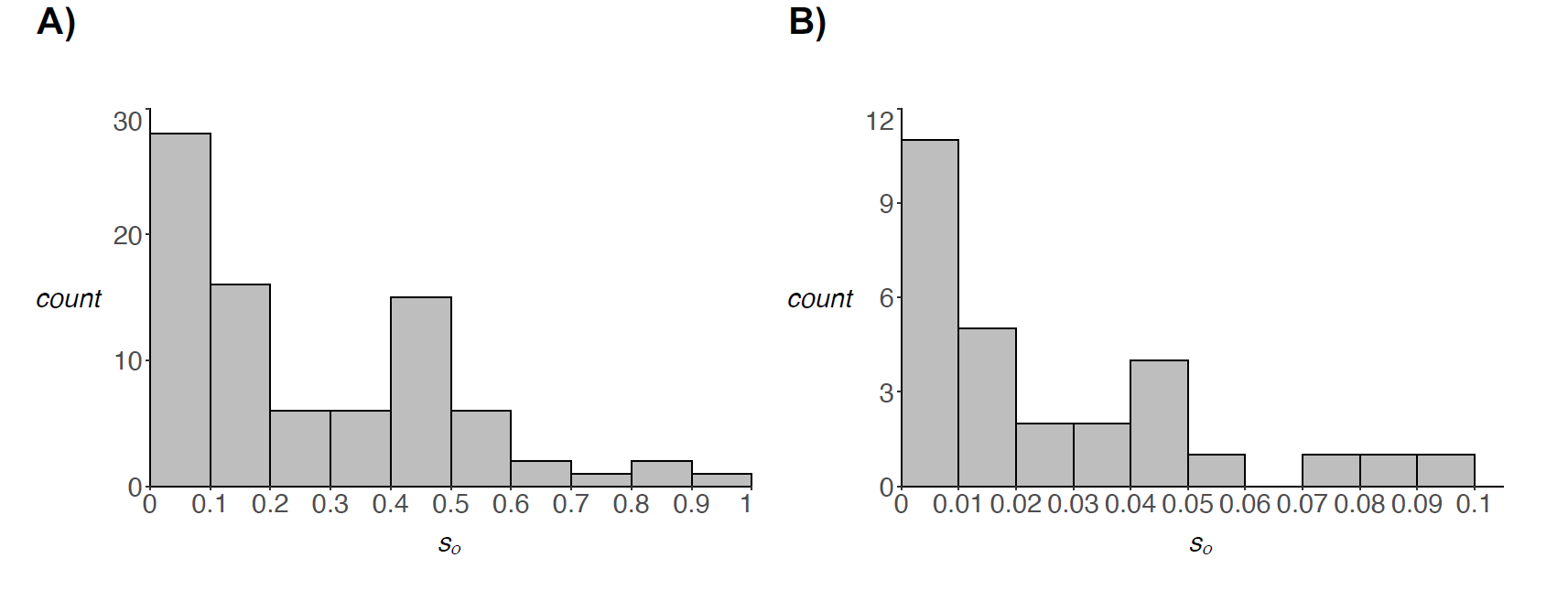


**Figure S2.** Distribution of overdominant coefficients (*s_o_*) found by Thurman and

Barrett (2016). (A) Full distribution; (B) Distribution of estimates with selection

coefficients lower than 0.1.

**Simulation of a structured population**

In the experimental setting (see main text), the bottles were numbered from 1 to 32. Over the whole period of 208 generations, in each generation half the individuals in bottle *i* were obtained from the corresponding bottle *i* of the previous generation and the other half from the previous one (*i* – 1), in turn.

We then simulated a panmictic population with *N_e_* = *N* = 10,000 individuals for 10,000 generations with a sudden decline in size to *N_e_* = *N* = 1,000 individuals occurred 208 generations in the past. Since that time, two scenarios were considered, one with a fully panmictic population, and another with 32 subpopulations with *N* = 32 individuals each, such that a migration rate *m* = 0.5 occurred every generation between consecutive subpopulations (1 with 2; 2 with 3 … 31 with 32; and 32 with 1).

The simulations were carried out with the software SLiM3 (Haller & Messer, 2019) assuming a nucleotide neutral mutation rate of 0.8e^–8^, and two chromosomes of 50 Mb of 0.5 Morgans each, with a recombination rate between consecutive nucleotides of 1e^–8^, i.e. 1 cM per Mb. Ten replicates were run for each of the two scenarios and the software GONE (Santiago et al. 2020) was used to estimate the historical *N_e_* in each replicate using a sample size of *n* = 100 individuals. For the structured population, 2-3 random individuals were taken from each subpopulation. The geometric mean of the estimates from the 10 replicates is shown in Figure S3.

As shown in the figure, when a fully panmictic population is considered (left graph), the estimation of *N_e_* by GONE reflects well the simulated population of 10,000 individuals (ancestral population), and the decline to a constant population size of 1,000 individuals 208 generation ago. However, if the population of 1,000 individuals is structured in subpopulation with 50% migration per generation between consecutive ones (right graph), the demographic changes in *N_e_* are estimated also rather precisely, but there is a sudden decline in the estimates of the most recent generations. This decline is an artefact of the method, because the simulated population was maintained with constant size over the 208 generations period.


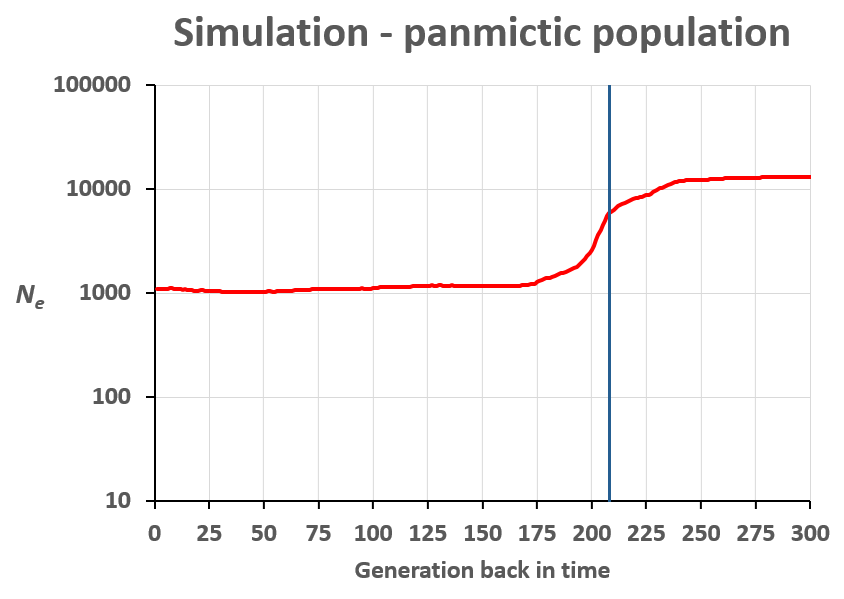

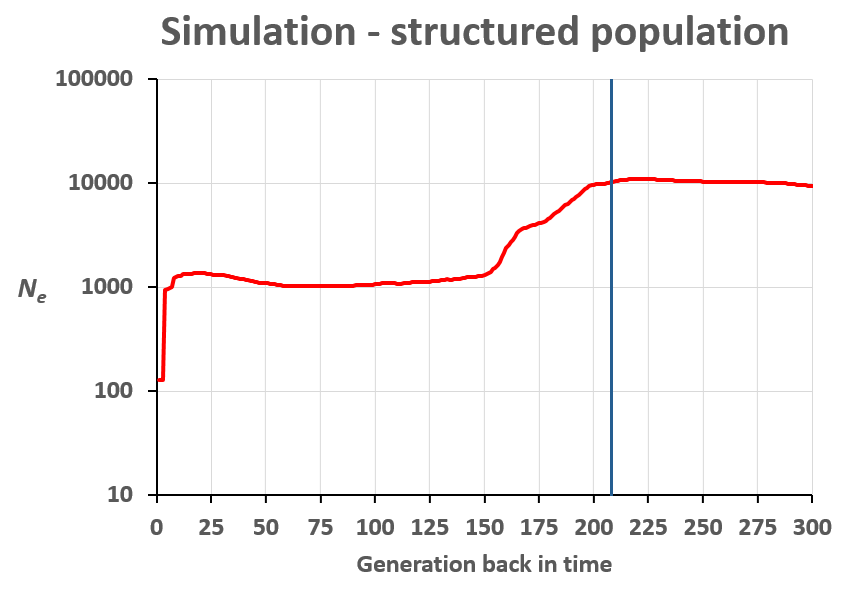


**Figure S3.** Estimated historical effective population size with the software GONE from simulation data. The lines represent the geometric mean of *N_e_* estimates based on 100 simulation replicates. The simulated *N_e_* was 10,000 before the foundation of a population of size *N_e_* = 1000 at generation 208 back in time (vertical line). The left panel refers to a fully panmictic population since generation 208, whereas the right panel refers to a structured population with 32 subpopulations of 32 individuals each, and 50% mixing between consecutive ones each generation.

**References**

Haller, B. C., & Messer, P. W. (2019). SLiM 3: forward genetic simulations beyond the Wright–Fisher model. *Molecular Biology and Evolution,* **36**, 632–637.

Santiago, E., Novo, I., Pardiñas, A.F., Saura, M., Wang, J., & Caballero, A. (2020). Recent demographic history inferred by high-resolution analysis of linkage disequilibrium. *Molecular Biology and Evolution*, **37(12)**, 3642-3653.
